# Health economic value of an innovation: delimiting the scope and framework of future market entry agreements

**DOI:** 10.3402/jmahp.v2.24988

**Published:** 2014-06-23

**Authors:** Robert Launois, Lucia Fiestas Navarrete, Olivier Ethgen, Jean-Gabriel Le Moine, René Gatsinga

**Affiliations:** 1 French Network for Evaluation in Health Economics, Paris, France; 2 Research Unit for the Economic Evaluation of Medical Innovation, University of Liege, Liege, Belgium

**Keywords:** risk sharing, pay for performance contract, comparative effectiveness research, cost-effectiveness studies

## Abstract

**Background and objectives:**

The objective of our paper is to offer a new, payer-friendly taxonomy of market entry agreements (MEAs) that aims to twin contracts with their methodological designs in an effort to clarify the distinction between contracts that are based on performance and those that are based on demonstrated effect.

**Methods:**

Our analysis proceeds in two stages: First, we delimit the scope and framework of pay for performance (P4P) and pay for demonstrated effect (P4E) agreements. Second, we distinguish the methodological designs supporting the implementation of each of these contracts.

**Results:**

We elucidate why P4P contracts prevent the payer from funding the true effectiveness of an innovation by expanding on their limitations. These include: 1) the normative nature of comparisons, 2) the impossibility of true effect imputability for each individual, and 3) the use of intermediary outcome measures. We then explore three main criticisms that payers must take into account when reasoning in terms of performance rather than in terms of the product effectiveness.

**Conclusion:**

The potential effect that performance-based reimbursements may have on dissociating the components of the cost-effectiveness ratio constitutes an obstacle to a true health economic reasoning.

Coverage of a new pharmaceutical product presents two major risks for the payer. The first originates from the uncertainty surrounding the actual benefit the product might deliver under optimal conditions of use. The second arises from the unpredictability of human behavior, including prescribers and patients. Any unexpected patterns of use might jeopardize the payer's ability to estimate a risk and establish effective counter measures to control it. As such, payers are exposed to a moral hazard. Various measures have been conceived in response to this problem. Among them, market entry agreements (MEAs) have attracted considerable attention in recent years across professional and academic circles (1). These agreements, also denoted as performance-based schemes (PBS) in Carlson and performance-based risk-sharing agreements (PBRSA) in Garrison, have been the subject of a call for a paradigm shift in drug reimbursement.

An MEA provides a contractual compromise when a payer is faced with considerable uncertainty about the effectiveness of a drug. The manufacturer is confronted by the opportunity cost of delayed market access while the society as such, and a patient in particular, faces delayed access to a potential therapeutic innovation. In such a situation, a drug is reimbursed on the condition that either 1) its performance be monitored or 2) its comparative effectiveness be evidenced. Performance is the degree of attainment of a predefined end point (i.e., tumor reduction), and comparative effectiveness is the added health benefit achieved under clinical routine practice with respect to a specific comparator. As such, there are two drastically different types of studies that may be put in place in order to satisfy the contractual conditions of an MEA.

The works by Carlson et al. (2) and Garrison et al. (3), in particular, have been central to the development of the current taxonomy on the reimbursement schemes of performance-based health outcomes. As such, two main categories of performance-based schemes have been identified: 1) conditional coverage, where coverage is conditional on starting a data collection surveillance program and 2) performance-linked reimbursement, where reimbursements are based on the degree of attainment of performance targets. Four additional subcategories emerge from these two schemes: 1) coverage with evidence development, where coverage is based on population-level evidence emanating from a scientific study, which can either be (a) only in research coverage or (b) only with research coverage, 2) conditional treatment continuation, where coverage is conditioned on the attainment of short-term treatment goals, 3) outcomes guarantees, where the manufacturer provides refunds or price adjustments according to treatment failure, which can either be based on: (a) clinical or (b) intermediate endpoints, and 4) pattern or process of care, where reimbursements are linked to the impact on clinical decision-making or practice patterns (2).

Our view is that the current literature explores the contractual nature of performance-based MEAs without sufficient clarity on the methodological design of the studies that are implemented to fulfill the conditions of such agreements.

The argument that we put forth is three-fold.

1. First, we believe the problem originates from the attempt to join two methodologically heterogeneous types of contracts under the same umbrella.
2. Second, we argue that the terminology used to describe the totality of the contracts as ‘performance-based risk-sharing’ might be misleading because it smoothens the differences between agreements based on performance and agreements based on comparative effectiveness. Moreover, the risk shared between the payer and the manufacturer is vastly different depending on the contracts signed between them.
3. Third, we warn against the widespread use of the term ‘performance’ in the literature, which is often assimilated with a greater level of evidence than it should warrant. The consequences of equating the performance of a drug with its attributable effectiveness can be catastrophic for a public system charged with assessing the real value of an innovation.

We believe that a clarification on these issues is important in order to have a more profound understanding of the scientific and population health implications of MEA contracts. We do so, while acknowledging that an information asymmetry exists between payers and manufacturers regarding the scientific rigor behind the contracts. The objective of our paper is, thus, to offer a new taxonomy of MEAs that aims to twin contracts with their methodological designs in an effort to clarify the distinction between contracts that are based on performance (P4P) and those that are based on demonstrated relative effectiveness (P4E).

1. First, we will delimit the scope and framework of P4P and P4E agreements.
2. Second, we will distinguish the methodological designs supporting the implementation of each of these contracts.
3. Third, we will explore the limitations of P4Ps based on the methodological quality of the descriptive studies that are used to satisfy the contractual attainment of performance.
4. Fourth, we discuss how performance-based reimbursements may dissociate the cost and effectiveness components of health economic reasoning.

## Scope and framework of MEAs, P4Ps, and P4Es

Prior to developing the proposed evaluation scheme in full, it is necessary to establish a common understanding of what we mean by MEAs. In this work, we use Adamski's definition of MEAs as ‘agreements concluded by payers and pharmaceutical companies to diminish the impact on the payer's budget of new and existing medicines brought about by either the uncertainty of the value of the medicine and/or the need to work within finite budgets’ (4). While this definition distinguishes between two MEA categories (i.e., health outcome based and financially based), the scope of this paper is delimited by a discussion of health outcome–based schemes, which may in turn impact the national health insurance budget.

Our proposed MEA taxonomy is designed with the three main decisions that a payer faces when drug pricing negotiations are at deadlock. As such, the payer may decide to enter a contract with the manufacturer that:

1. Rewards performance
2. Penalizes underperformance
3. Earmarks funds to obtain evidence

If the payer's decision is based on ‘performance’, the contracts fall under the family of pay for performance (P4P). The main characteristic of these contracts is that the payer and the manufacturer agree on a clinical target to be used as a threshold assessing binary performance (i.e., the drug works or it does not).

### Contracts that reward performance

If the payer enters a contract that rewards performance, it reimburses the manufacturer on a case-by-case basis for each individual patient, each time the product meets the target. Contracts such as pattern/process of care (PPC) and conditional treatment continuation (CTC) belong to this category. Though slightly different in their definition of performance, both PPCs and CTCs are based on reimbursements conditioned on meeting a prespecified target. In the case of a PPC, the performance target is, for example, patient adherence to a suggested treatment line, whereas, in the case of a CTC, performance is based on the attainment of short-term treatment goals (2). In practice, the payer may reimburse the manufacturer each time a patient complies with the suggested treatment (i.e., PPC) or for as long as the drug delays disease progression (i.e., CTC).

### Contracts that penalize underperformance

Likewise, if the payer enters a contract that penalizes underperformance, it agrees to reimburse the amount requested by the manufacturer on the condition that the manufacturer offers a refund each time the drug fails to meet the target. This is the case for contracts such as outcomes guarantees (OG), where the manufacturer provides rebates, refunds, or price adjustments should the product underperform (2).

### Contracts that earmark funds to obtain evidence

Conversely, if the payer's decision is based on ‘evidence’, there is one pay for demonstrated relative effectiveness (P4E) contract to consider: coverage with evidence development (CED). Under a CED, the payer earmarks funds for the manufacturer to conduct the appropriate relative effectiveness and efficiency research guiding reimbursement decisions for the entire research population (i.e., coverage *in* research) or the entire population of concerned patients (i.e., coverage *with* research) (2, 3). The payer gives the manufacturer a subsidized time-window to reveal the true clinical and/or medico-economic usefulness of the product. The main difference between this contract and the P4P family (i.e., PPC, CTC, and OG) is that the goal of a CED is to demonstrate the direct and exclusive attributable effect that a given product has on patient health at the population level (5). As such, a CED contract is the most scientifically rigorous route, albeit not favored by manufacturers due to the direct costs of collecting effectiveness and efficiency evidence (3).

## Methodological designs supporting the implementation of MEAs

The main critique of MEAs is that the vast majority of the reimbursement schemes are not supported by rigorous methodological designs. The question that P4P contracts consider can be summarized as *Does the drug perform according to the expected value agreed upon between payer and manufacturer?* This is a significantly less complex question to address than that considered by P4E contracts, which can be translated as: *Does the drug have a direct and exclusive effect on the patient's outcome?* However, contracts based on performance outnumber those based on demonstrated effect proving that the former are easier to implement. Thus, it must be acknowledged that the majority of MEA contracts reward the manufacturer with pricing flexibility based on normative (i.e., contractual) rather than scientific (i.e., causal) standards.

**Fig. 1:**
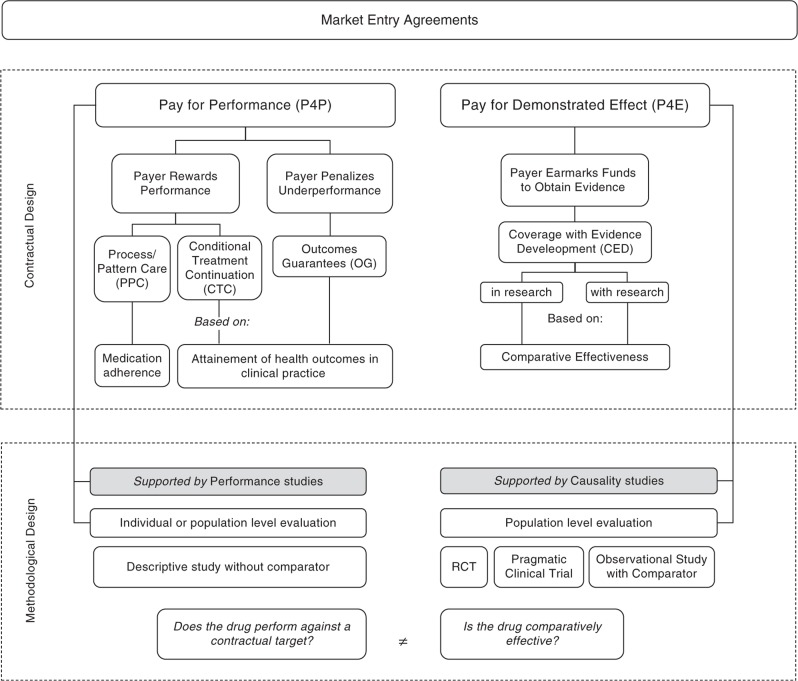
New payer-friendly taxonomy of market entry agreements.

Enthusiasm surrounding the pricing flexibility that characterizes P4P contracts has shadowed the methodological deficiencies that are inherent when hypothesis-testing is based on the performance rather than the attributable effectiveness of a product. As such, it is important to highlight the differences in methodological rigor between P4P and P4E contracts. The former must be twinned with the normative study design that enables its performance-based hypothesis-testing, henceforth denominated as *performance study*. The latter should be matched with the causal design permitting the evaluator to test hypotheses based on effectiveness/efficiency, hereafter denominated as *causality study* (Fig. 1).

## Limitations of P4P contracts and performance studies

A performance study within a P4P agreement aims to ensure that the commitments made by manufacturer and payer are fulfilled. Such audit-like design may be conducted in relation to a medico-administrative or an economic criterion. There will be discordance between the expected and the observed result because the external performance reference is built on findings from controlled trials, while performance itself is confounded by uncontrolled real life factors. The payer and the manufacturer might be able to set up a performance threshold based on clinical trial results. Yet, the uncertainty of translating knowledge originating from a controlled clinical trial environment into the standard to be expected in clinical practice could subject pricing decision-making to pure chance. Moreover, P4P evaluations do not allow the payer to understand:

1. How and why a treatment could have interrupted natural disease progression.
2. Whether the observed changes in the patient's health are directly and exclusively due to the administration of the product.
3. Whether the innovative product is comparatively effective vis-à-vis alternative treatments.

We proceed to elucidate why performance-based contracts prevent the payer from funding the true effectiveness of an innovation by expanding on their limitations. These include 1) the normative nature of comparisons, 2) the impossibility of true effect imputability for each individual, and 3) the use of intermediary outcome measures.

### Normative nature of comparisons

The ‘performance’ approach to drug pricing leans toward the ‘normative research’ definition that Contandriopoulos (6) and the US General Accounting Office (7) refer to as: ‘a judgment made based on the comparison of the used resources, services rendered or goods produced, and obtained results with certain norms and criteria’. In Gridchyna's doctorate work regarding the use of juridical norms as regulatory market access instruments, the author argues that although norms are negotiated through a contractual agreement between the industry and the state, this does not alter their legal and unscientific content (8). In the P4P context, the payer is more preoccupied about the proper use of an innovation rather than how useful it actually is.

By measuring performance, a treatment could reach the preestablished target value, but the true effect of the treatment may not be the cause behind the value attained, rendering performance-based evaluations unreliable. In this vein, an evaluation becomes ‘virtual’ in every respect except for the legal aspects of the commitments made. To estimate the ‘true result’ that can be attributed solely to the treatment under consideration, the decision maker needs more robust analytical tools. When using P4P contracts, the uncertainty surrounding the demonstrated effectiveness/efficiency of a treatment remains. The negotiated and observed performance targets may be equal, without elucidating whether this equality is directly and exclusively attributed to the treatment.

### Impossibility of true effect imputability for each individual

Evaluating a causal relationship between a treatment and a health outcome in an individual necessitates a direct comparison between the treatment-related benefit gained by the individual under treatment A and the treatment-related benefit that the same individual would have gained should she/he have taken a different treatment (9, 10). This is the principal canon justifying the use of pragmatic clinical trials, followed by observational studies with post-hoc micro-econometric corrections in order to assess a causal link between treatment and effect. Through design, these studies recreate a counterfactual for each participant and, in so doing, isolate the true treatment effect from the effect of confounders. While it may be argued that the external validity (i.e., generalizability) of the innovation is an important aspect for payers, it is widely acknowledged that when considering the trade-offs between internal validity and generalizability, validity is to be preferred as a matter of good practice. Schneeweiss argues: ‘From a clinical perspective, the most important generalization to make is about the comparability of therapies among patients for whom either of the drugs would be a reasonable treatment choice. Comparative-effectiveness research among these patients may not yield the most generalizable information, but it will yield information that is most relevant to clinical decision-making’ (11).

Performance studies, however, do not envision a counterfactual in their design, posing a problem for P4P agreements where a drug is reimbursed on a case-by-case basis. Unlike P4E, in a P4P contract, the payer is charged each time the target outcome is reached. However, the payer does not know the true contribution of the drug in attaining the outcome given the inherent lack of comparative evidence in the design. When reimbursing each individual performance target attained, the true effectiveness of the product in question cannot be known due to the absence of a counterfactual. In this regard, a drug is thought to have ‘worked’ although there is no way to verify whether the patient's improvement is due to the administration of the drug or due to other reasons. In fact, the payer rewards the manufacturer while remaining ignorant about the true effect that the drug may (or may not) have on the health outcome.

A counterfactual situation cannot be estimated without calculating the total treatment difference in effectiveness between the average health outcome of patients treated with the innovative treatment and the average health outcome of patients treated conventionally. In other words, the effect attributable to the treatment must be completely isolated from the effect of the confounding covariates in order to make a causal inference. Giving P4P's inability to reveal the true effect of a product, the payer is obliged to rely on low-quality evidence, which may, at best, reveal an association unconditioned for confounders.

### Use of intermediary outcome measures

When a P4P reimbursement commitment uses an intermediary endpoint as the external performance target, as most OG contracts do, an additional problem arises. Ideally, if a reimbursement decision is based on intermediary performance targets, this target should show a strong correlation with the clinical parameters that it intends to replace. Only then, can the intermediary target be considered a valid surrogate criterion. In most cases, defined intermediate endpoints do not necessarily lead to clinical endpoints. For instance, in cancer studies, the correlation between the progression-free survival and the global survival is not well established. In the same way, diagnoses made using biomarkers are hindered by the scarcity of clear-cut predicting markers. When using biomarkers, the resulting sensitivity, specificity, and predictive values assessing test positivity are mediocre at best (12). As such, biomarkers may lead to false-negative and false-positive results.

If one adds the outcome measurement risk of using intermediary performance endpoints to the known problem of individual imputability, the evaluation moves further away from scientific rigor. The evaluator is then unable to assess the true effectiveness of a health outcome (due to the lack of a counterfactual), and perhaps more seriously, he/she risks choosing an intermediary performance target that does not represent the health outcome that the drug is sought to effect.

## Discussion

When the objective of the assessment is to establish a causal link between drug and health outcome, and the ambition of the payer is to reimburse drugs that are shown to be directly and exclusively effective when managing a health condition, a causality study must ensue. This is the methodological turf for reimbursements based on P4E contracts. Having elucidated the methodological limitations surrounding the use of P4P contracts, we explore three main issues that payers must take into account when reasoning in terms of performance and at the expense of product effectiveness or efficiency. We conclude by reflecting on the foreseeable consequences of disfiguring the economic value of an innovation through an over-simplistic understanding of product performance.

### Performance is not evidence

Consider the risk of confounders in a performance study that, at best, compares an observed target against a contractual target without accounting for potential confounders. In the absence of confounder control, risks for bias rise. Putting aside the fact that a study design without a counterfactual is incapable of assessing true treatment effectiveness, a most severe issue is the evaluator's ignorance vis-à-vis the existence of any and all unobserved factors affecting drug performance. In a performance study, the drug is one of the many unobserved factors affecting the performance target. As such, we should not mistake the findings of a performance study as ‘evidence’ of drug performance but as a ‘result’ emerging from an uncontrolled environment. These performance results must be interpreted in light of the crippling limitations inherent in the study designs that produced them. Performance, in this sense, is but the observed consequence following treatment administration independently of whether or not the treatment is the actual cause. As such, performance results may not be equated to evidence of true drug effectiveness.

Performance can be explained as the administrative task of assessing whether external references are met. This implies that a closer look at the actual magnitude and significance of the therapeutic effect is foregone. Measuring performance does not capture the real value of an innovation and is an inappropriate response to the demands of evidence-based decision making. As an indicator, performance merely assesses whether a recognized outcome level has been dully attained but does not provide insight on the real health effect of the innovation. As such, performance measures offer a set of informed assumptions *with reference to* health outcomes but *not directly or exclusively linked to* health outcomes. There is a tectonic difference, if only subtly misplaced by vocabulary. Should we be interested in applying econometric rigor to performance, one may realize that the attainment of ‘targets’ bears no reflection on the certainty that they are in fact the direct effect of the innovation.

### Payers are not tasked to reason as patients

The ultimate impact of the performance chain corresponds to the one and only true result in the patient's eye: *When I take the medication, do I get better or not?* While this may be an objective that is shared both by the patient and the payer, the latter is also responsible for understanding the significance and the magnitude of the product's effect on the health condition. Most importantly, the payer is accountable for funding a product whose effect can be reproducible to the whole population of patients. When the system reasons in terms of performance, neither the patient nor the payer is able to appreciate how much of the health improvement is due to the drug and how much due to his/her clinical history, age, level of physical activity, or any other confounding factor. Moreover, performance does not inform the payer if a cheaper option would have performed as well; thus, it does not address proper use of resources in a scarce environment.

The patient can put together an illness narrative where the drug is responsible for the improvement. The payer, on contrary, is tasked with the health of a population. As such, the generalizability of the results obtained should be of upmost importance. The payer must care to know whether the drug is the protagonist of the health effect incurred in a population of patients. Moreover, it must understand how much of the health improvement is directly and exclusively due to the use of the drug. This is not possible when the payer thinks in terms of performance because neither the internal nor the external validity of the results can be guaranteed.

When payers are encouraged to reimburse on an individual patient basis, they forego their population-based responsibility to verify treatment–effect causality. P4P contracts then imply a reductionist view of the payer's accountability to population health. It follows that by failing to reward the actual attributable effectiveness of a treatment, reimbursements that are made on drug performance are fiscally imprudent.

### The consequence of divorcing cost from effectiveness

In countries where a willingness-to-pay (WTP) threshold defines the socially acceptable cost of introducing health products, drug performance may be evaluated through the use of cost-effectiveness ratios (i.e., how many euros per year of life gained?). If the new drug costs less than the socially accepted ‘euros per unit of health’ threshold, the payer reimburses the manufacturer, if it costs more, the payer does not. However, in countries where innovations are not reimbursed based on a cost-effectiveness WTP threshold, performance thresholds lead to intuitive decision-making based solely on clinical parameters. While a cost-effectiveness WTP threshold contains information both on the cost and on the effectiveness of an innovation, a P4P threshold reduces an evaluation to absolute product performance. Evidently, public health economic thinking is lost from the moment the payer bases its WTP on contractual clinical thresholds.

A paradigm shift toward performance-based reimbursements threatens to dissociate the components of health economic reasoning. Costs will be progressively studied in isolation from full medico-economic evaluations as they become the subject of cost-sharing arrangements (2, 3). In this way, costs will be understood in their narrowest accounting sense and the health economic value of an innovation will be replaced by its absolute cost to the health system. As stated by Garrison, an analysis of costs will inform decisions made based on ‘cost-sharing arrangements’, and an analysis of clinical parameters will inform ‘risk-sharing arrangements’ (3). In the absence of an acceptable cost-effectiveness WTP threshold, the added value of an innovation will need to be understood exclusively in clinical or in cost terms. Value, which can only be appreciated with paralleled cost and effectiveness lenses, will go out of the window.

## Conclusion

We advance that P4P agreements present a disproportionately risky move for the payer, given that reimbursement commitments are not based on the true value of an innovation. We foresee that performance-based contracts will become a political instrument to guard payers from the criticism that results when the reimbursement of a potentially life-saving product is denied. Thus, there is an emerging need to deepen methodological research on this topic in order to enable our discipline to adequately respond to the demands of public authorities.
